# Children with developmental coordination disorders: a review of approaches to assessment and intervention

**DOI:** 10.3389/fneur.2024.1359955

**Published:** 2024-05-23

**Authors:** Jiaxin Gao, Wei Song, Yue Zhong, Dunbing Huang, Jiaqi Wang, Anren Zhang, Xiaohua Ke

**Affiliations:** ^1^School of Health Preservation and Rehabilitation, Chengdu University of Traditional Chinese Medicine, Chengdu, China; ^2^Department of Rehabilitation Medicine, Shanghai Fourth People's Hospital, School of Medicine, Tongji University, Shanghai, China

**Keywords:** developmental coordination disorder, rehabilitation, international classification of functioning, disability and health, child, physical and rehabilitation medicine

## Abstract

Developmental Coordination Disorder (DCD) is a neurodevelopmental disorder characterized by deficits in motor skills, with gross and fine motor dysfunction being the main symptom. This condition greatly impairs children’s daily life, learning, and social interaction. Symptoms typically appear during preschool or school age, and if left untreated, they can persist into adulthood. Thus, early assessment and intervention are crucial to improve the prognosis. This study aims to review the existing literature on DCD, providing a comprehensive overview of the assessment for children with DCD in terms of body functions and structures, activities and participation, and environmental factors within the framework of the International Classification of Functioning, Disability, and Health - Children and Youth (ICF-CY). Additionally, specific rehabilitation interventions will be described, offering valuable insights for the clinical assessment and intervention of children with DCD.

## 1 Introduction

Developmental Coordination Disorder (DCD) is a neurodevelopmental disorder that impairs a child’s ability to perform coordinated motor movements, leading to slow, clumsy, or inaccurate movements and difficulties in learning new movements (1). However, it is important to note that DCD is not caused by organic, intellectual, or psychological issues (2), but instead by irregularities in the brain areas responsible for processing motor information (3). The prevalence of DCD in children ranges from approximately 2 to 20%, with widely accepted international figures currently standing at 5–6% (4). DCD is characterized by deficiencies in both gross and fine motor skills. Children with DCD often exhibit clumsiness, lack of balance and coordination, and struggle with everyday tasks such as grasping objects, dressing, and writing. Additionally, DCD is frequently associated with other conditions such as developmental language disorders, attention deficit hyperactivity disorder, autism spectrum disorders, learning disabilities, and cognitive deficits (4–9).

Furthermore, limited mobility stemming from motor skill deficits can result in decreased opportunities for children with DCD to engage in sports or social activities. This, in turn, increases the risk of weight issues or obesity and may lead to social deficits like isolation, reduced participation in social activities, and difficulties in forming friendships (10–12). Moreover, individuals with DCD may also experience psychosocial challenges such as anxiety and depression (13, 14). These psychosocial problems can persist into adulthood and significantly impact adaptive behavior and mental health outcomes (15). Hence, early diagnosis and intervention are crucial in improving long-term prognosis for individuals with DCD.

The International Classification of Functioning, Disability and Health for Children and Youth (ICF-CY) is a comprehensive framework that categorizes human functioning and disability. It serves as a scientific tool to understand and explore health-related states, outcomes, and factors that influence them (16). The ICF-CY encompasses three main components: body functions and structures, activities and participation, and environmental factors (4, 16). Body functions and structures refer to the anatomical parts and physiological functions of the body systems. Activities pertain to the individual’s ability to perform tasks or actions. Participation relates to the individual’s engagement in various life situations. Environmental factors encompass the natural, social, and attitudinal aspects of people’s surroundings that influence and interact with the elements of body functions and structures, activities, and participation.

This review examines the assessment of children with DCD within the context of the ICF-CY framework. It provides an overview of how children’s functioning in terms of body functions and structures, activities and participation, and environmental factors is evaluated. Additionally, specific rehabilitative interventions are discussed to offer insights into the clinical assessment and treatment of children with DCD.

## 2 Assessment of children with DCD

The assessment of DCD is underpinned by a variety of theoretical frameworks, including the following five (4, 17): Firstly, there’s the functional motor skill assessment, which relies on normative standards such as MABC-2, BOT-2, and other standardized tests designed to evaluate skill performance. Secondly, there’s assessment based on general ability, which concentrates on the child’s sensory-motor integration, encompassing factors like perceptual ability. Thirdly, there’s assessment rooted in neurodevelopmental theory. This approach examines the child’s neurological function and signs, including various hard and soft indicators. Fourthly, there’s the dynamical system-based assessment, which delves into biomechanical and kinematic analyses to understand motor function dynamics. Lastly, there’s the cognitive-neurological based assessment, which focuses on evaluating the brain’s functioning concerning motor skills. This review aims to provide a multilevel clinical assessment of children with DCD, drawing from the above assessment theories to comprehensively reflect motor development across different functional levels—behavioral, neurocognitive, and affective.

### 2.1 Clinical diagnosis of DCD

**Figure 1:**
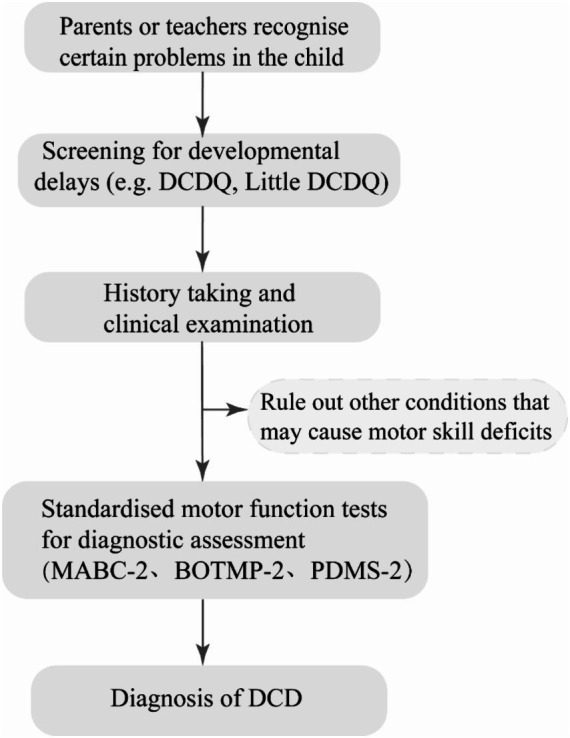
Clinical diagnosis of DCD. DCDQ, Developmental Coordination Disorder Parent Questionnaire; MABC-2, Movement Assessment Battery for Children, Second Edition; BOTMP-2, Bruininks - Oseretsky Test of Motor Proficiency, Second Edition; PDMS-2, Peabody Developmental Motor Scales, Second Edition; DCD, Developmental Coordination Disorder.

Clinical assessment and diagnosis of DCD involves various steps, including screening, history taking, clinical examination, and standardized diagnostic assessment (see Figure 1) (4). DCD is typically identified by parents or teachers when a child shows signs of delayed motor development, difficulties with coordination and daily activities. Screening questionnaires like the Developmental Coordination Disorder Parent Questionnaire (DCDQ) and Little DCDQ are commonly used to identify children with DCD based on parent reports, assessing motor control, fine and gross motor skills, and overall coordination. Once children are screened positive, a history and clinical examination are conducted to rule out other conditions that could be causing motor skill deficits. If no other condition is identified, further diagnostic assessments are carried out using standardized tests like the Movement Assessment Battery for Children, Second Edition (MABC-2), Bruininks - Oseretsky Test of Motor Proficiency, Second Edition (BOTMP-2), and Peabody Developmental Motor Scales, Second Edition (PDMS-2). The MABC-2 is the most widely used and well-studied test for diagnosing DCD, evaluating fine motor, gross motor, and balance skills. The BOTMP-2 assesses upper limb movement quality, coordination, balance, and bilateral coordination (4). On the other hand, the PDMS-2 assesses gross and fine motor development in young children and is commonly used for assessing motor function in various disease states (18). It is important to note that while the MABC-2 is considered the gold standard for assessing motor development, a single motor test may miss some cases of DCD. In such instances, the BOTMP-2 can be performed simultaneously to avoid missed diagnoses. These assessment tools are utilized by occupational therapists and physiotherapists to observe and evaluate a child’s performance on specific motor tasks. Table 1 summarizes the test category, applicable conditions, quality, strengths, limitations and the level of evidence for guideline (4) recommendations of commonly used assessment scales for DCD.

**Table 1:** Clinical standardized assessment tools.

Assessment tools	Test category	Applicable conditions	Quality	Strengths	Limitations	Recommendation level
DCDQ	Motor control, fine motor skills/writing, gross motor/planning skills, overall coordination	5 ~ 15 years	Good reliability; good validity	Good level of evidence and the most widely used subjective screening tool for motor development problems in children	For screening for DCD, not for diagnostic tests for DCD	Good
MABC-2	Manual dexterity; aiming and catching; balance	3 ~ 16 years	good to excellent reliability; fair to good validity; good specificity	The most used and best studied standardized movement test for individual diagnosis of DCD	Slightly lower sensitivity; obesity may affect test results	Moderate to good
BOTMP-2	Fine motor accuracy, fine motor integration, dexterity, bilateral coordination, balance, running speed and agility, upper body coordination, strength qualities	4 ~ 21 years	good to excellent reliability; fairly good validity; good specificity	Assesses a wide range of motor skills in individuals and is applicable to a wide age range	Highly difficult test items, complex and time-consuming evaluation and scoring, high demand on testers and test space	Moderate
PDMS-2	Reflexes, posture, movement, substantive manipulation, grasping, visuomotor integration tests	0 ~ 5 years	good reliability; moderate validity	Commonly used to assess motor function in children in a variety of disease states and to assess the presence of motor developmental delay	Scale applies to younger ages and has not been validated for DCD diagnosis	Low

### 2.2 ICF-CY – based assessment

**Figure 2:**
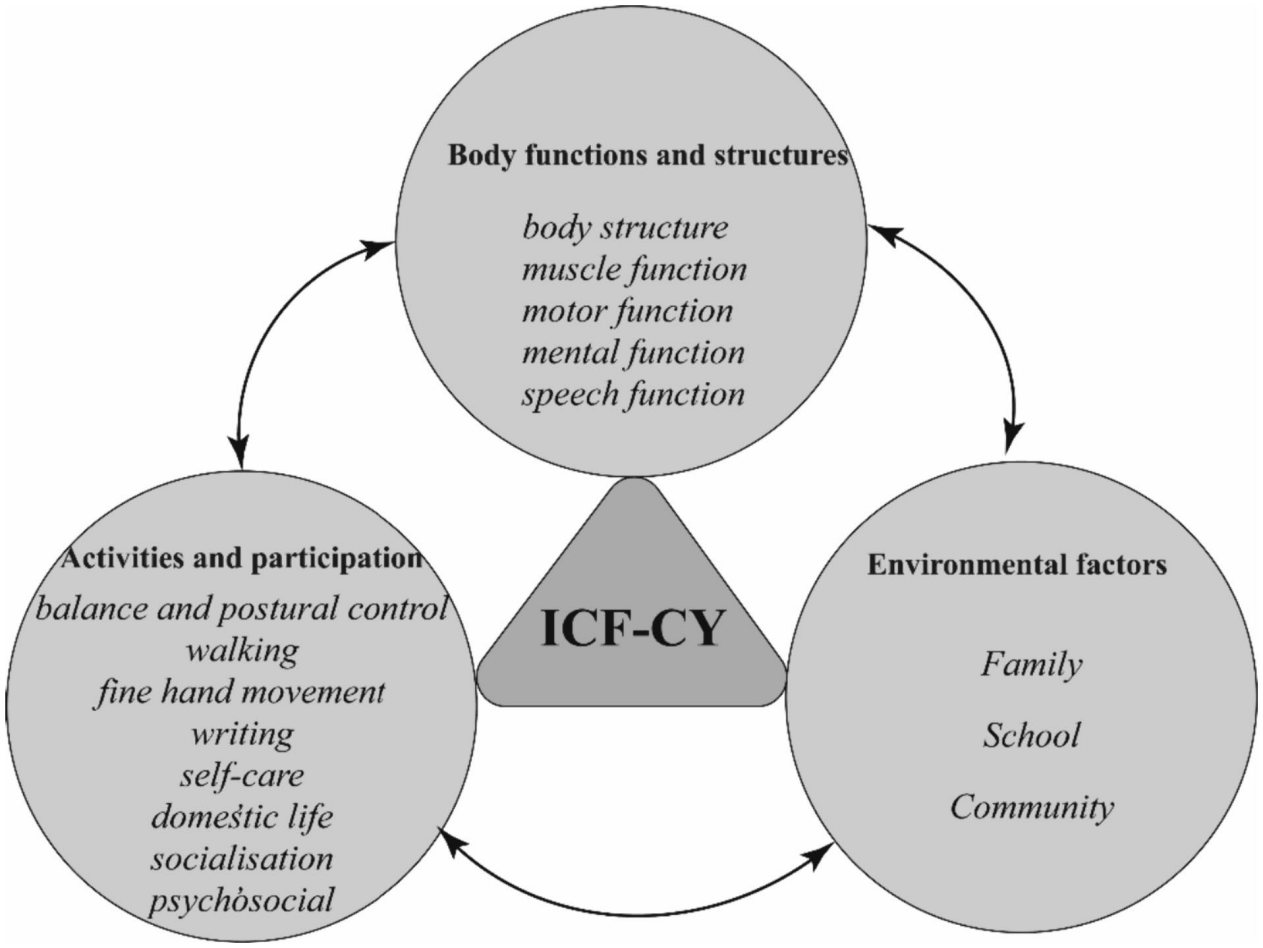
Rehabilitation assessment of children with DCD under the ICF framework. ICF-CY, International Classification of Functioning, Disability and Health for Children and Youth; DCD, Developmental Coordination Disorder.

The assessment of children with DCD should not solely focus on standardized scales, but should take a holistic approach within the framework of the ICF in the field of rehabilitation medicine. In addition to evaluating impaired body functions and structures, it is crucial to assess restricted activities and participation, as well as consider environmental factors that may impact a child’ s functioning (see Figure 2).

#### 2.2.1 Assessment of body functions and structures

The assessment of body functions and structures in children with DCD involves evaluating various aspects such as body structure, muscle function, motor function, mental function, and speech function (see Table 2).

**Table 2:** ICF-CY - based assessment.

ICF-CY – based assessment	
Assessment of body functions and structures	Assessment approaches
Body structure	
Body posture	
Joint activity	
Skeletal development	
Body mass index	
Muscle function	
Muscle strength	
Muscle tone	
Motor function	
Reflex function	Deep tendon reflexes; plantar reflexes; adductor reflexes
Primary reflexes	Retraining for Balance
Motor control	Finger-nose test; finger-finger test; alternating movements
Mental function	
Intelligence	Wechsler intelligence scale for children
Advanced cognitive function	
Executive function	
Working memory	Digit span test; Groton Maze Learning Test; Block tapping test
Inhibitory control	Flanker task; Go/NoGo test; Stroop test
Cognitive flexibility	Wisconsin card sorting test
Motor planning	“End-state-comfort” effect
Perceptual function	
Tactile perception	Tactile discrimination task
Visual perception	Test of Visual-Perceptual Skills (TVPS-4)
Visuospatial perception	Visual imagery task Mental rotation task
Visuomotor skills	Visual-motor integration test; hand-eye co-ordination; replicated shapes; three-dimensional (3D) block designs; 3D motion capture techniques
Speech function	
Verbal expression (phonological errors, syllable separation, rhythm abnormalities, and poor coordination of oral motor skills)	Assessment of maximal vocal duration, counting ability, oral rotation rate, and speech articulation
Verbal comprehension	Wechsler intelligence scale, fourth edition MacArthur communicative development inventory
Assessment of activities and participation	
Balance and postural control	Functional balance tests Berg balance scale; Timed up and go test; Timed up and down test; Limit of stability; Functional forward extension tests Balance test system
Walking (kinematics, kinetics, and plantar dynamic during walking)	Coronal 2D video recordings and posture estimation; 3D motion capture system
Fine hand movement	Fine motor assessment system 3D motion capture
Writing	Standardized scale (handwriting proficiency screening questionnaire; detailed assessment of speed of handwriting scale)
Handwriting legibility	Handwriting legibility scale
Handwriting-generated movement	Digital writing tablet; 3D motion technology
Performance and engagement in ADLs	DCD daily scale
Social	Social skills improvement system questionnaire
Psychosocial	Strengths and difficulties questionnaire
Participation	Child participation scale; Canadian occupational performance measure
Assessment of environmental factors	
Family environment	Personality traits of family members; living conditions; lifestyle; exercise spaces; parental attitudes and behaviors
School environment	Availability of space and facilities; classroom activities and interaction; school adjustment, peer relationships, and teacher–student relationships
Community environment	Outdoor sports spaces; playmates; community activities

**Figure 3:**
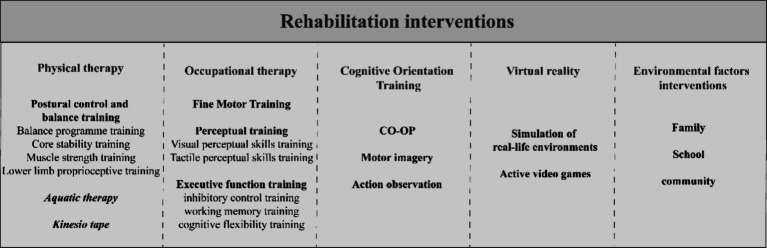
Clinical rehabilitation interventions for children with DCD. CO-OP, cognitive orientation to daily occupational performance; DCD, Developmental Coordination Disorder.

Assessment of body structure and muscle function primarily revolves around evaluating the power system. An examination of the body structure looks for abnormal body posture, joint hypermobility, swelling or pain in the joints. Additionally, due to restricted activities and limited participation, children with DCD may encounter delays in skeletal development (19). Thus, it’s essential to screen them for skeletal maturity, considering their individual circumstances, including body mass index (BMI). Additional measurements and analyses, such as arm span and leg length, should be conducted if deemed necessary. Muscle function tests assess muscle tone and strength, which are often found to be low in children with DCD (20, 21). Motor function was assessed based on neurodevelopmental theories, including evaluations of motor reflex function, primary reflexes, and random motor control function derived from the dynamical system perspective. Children with DCD process motor reflexes differently than their peers, and reflexes like deep tendon reflexes, plantar reflexes, and adductor reflexes can be examined (22). Posture development, which relies on the integration of primary reflexes, is a prerequisite for movement (23). The Niklasson study (24) noted that unintegrated primary reflexes and vestibular dysfunction contribute to sensorimotor developmental delay and are important aspects for diagnosing DCD. Niklasson assessed primary reflexes and vestibular function by means of Retraining for Balance (RB), the RB-Physiological test detects abnormal primary reflexes, especially those related to the vestibular system, such as asymmetric tonic cervical reflexes, tonic labyrinth reflexes, symmetric tonic cervical reflexes, and Morrow’s reflexes (24), RB-Orientation and balance tests are used to detect vestibular function in children (25). However, international clinical guidelines (4) published by the European Academy of Childhood Disability (EACD) state that the role of mild cerebral developmental disorders and mild neurological dysfunction in the diagnosis of DCD is controversial. Therefore, the value of signs of abnormal motor development in the diagnosis of children with DCD needs to be verified by further studies. In terms of random motor control function, hand-eye coordination is usually poor in children with DCD, and tests such as the finger-nose test, finger-finger test, and alternating movements can be performed to assess coordination function (Figure 3).

Assessment of mental functioning consists primarily of intelligence and advanced cognitive functions (executive functioning and motor planning) based on cognitive neurological assessment, and perceptual functioning based on general abilities. The Wechsler Intelligence Scale for Children is utilized to evaluate the children’s intellectual functioning (26). Poor executive functioning is also found in 40–60% of DCD cases (3, 27), including the three main areas of working memory (especially visuospatial working memory), inhibitory control and cognitive flexibility (27). Working memory can be assessed using the digit span test (28, 29), and visuospatial working memory can be assessed using the Groton Maze Learning Test (GMLT) and the block tapping test (30). Multiple test tasks such as the Go/NoGo test (31), Stroop test (32, 33), Tower of London test (31), etc. can be used to assess executive functions such as motor inhibition, cognitive inhibition, spatial problem solving and planning tasks. Moreover, children’s inhibitory control can be assessed by objectively recording reaction time and accuracy while individuals complete non-conflict and conflict tasks using the Flanker task (34). Longer reaction times and lower accuracy typically indicate poorer attentional inhibition. The Wisconsin Card Sorting Test (35) evaluates children’s cognitive flexibility and directly assesses their ability to think abstractly by reasoning analogically about sorting and matching cards. Recorded variables include categories completed, correct responses, total errors, perseverative errors, non-perseverative errors, and conceptual level responses. Motor planning was assessed by the “end-state-comfort” effect in the grip task, i.e., whether the selection of the object to be grasped was prioritized so that the movement could end comfortably (36). Assessment of perceptual function should focus on tactile perception and visual perception, previous studies have found that motor deficits in children with DCD are associated with tactile perceptual dysfunction, and that children with DCD have reduced tactile acuity compared to normal children (37, 38), a tactile discrimination task can be used to examine children’s tactile acuity. In terms of visual perception-related functioning, focusing on visuospatial perception as well as assessment of visuomotor skills, research studies have found that 5–47% of children with DCD are impaired on visual or visuospatial perception measures and 5–42% on visuomotor measures, visual perception plays a key role in the development of motor skills, and low functioning can lead to motor deficits (39). Therefore, identifying visual perception deficits is particularly important in assessing DCD. The standardized scale Test of Visual-Perceptual Skills-Fourth Edition (TVPS-4) provides a comprehensive test of visual-perceptual skills, The Visual Imagery Task and the Mental Rotation Task (40) assess visuospatial perception, involving tasks such as identifying three-dimensional shapes in various spatial orientations or angles through observation and imagination. Visual-motor skills can be assessed through the Visual-Motor Integration Test, hand-eye co-ordination (e.g., finger-nose test), replicated shapes, and three-dimensional block designs (26, 41). In addition, three-dimensional motion capture techniques are increasingly being used in the perceptual assessment of children with DCD, Wilmut (42) used digital calipers to detect DCD children’s judgement of the amount of space they could pass through a gap to assess the children’s perceptual bias and found that the children in the DCD group overestimated the amount of space required to pass through the gap (42).

Lastly, the development of motor skills can impact speech and language skills (43). Specific speech disorders, including phonological errors, syllable separation, rhythm abnormalities, and poor coordination of oral motor skills, are often observed in children with DCD (44). Assessments for speech include measures like maximal vocal duration, counting ability, oral rotation rate, and speech articulation. Additionally, it’s crucial to incorporate measures of verbal comprehension, utilizing specific tools like the verbal test, vocabulary test, reading fluency test, and reading comprehension test from the Wechsler Intelligence Scale, Fourth Edition (45). Other assessments to consider include words understood and words used in the MacArthur Communicative Development Inventory – words and gestures (MCDI) (46).

#### 2.2.2 Assessment of activities and participation

From a limitations perspective, the assessment of activities and participation of children with DCD includes a variety of areas such as balance and postural control, walking, fine hand movement, writing, self-care, domestic life, socialization, and psychosocial aspects (see Table 2). The assessment primarily relies on the dynamical system approach, supplemented by cognitive neurology.

Balance and postural control, which involve the ability to control the body in static and dynamic situations, can be assessed through both non-instrumented and instrumented tests (47). Non-instrumented tests mainly include functional balance tests such as the Berg Balance Scale, Timed Up and Go Test, Timed Up and Down Test, Limit of Stability and Functional Forward Extension Tests (48). However, these non-instrumented tests only provide a qualitative evaluation. On the other hand, instrumented tests, like the Virtual Reality Rehabilitation System (VRRS), can provide quantitative analysis of postural control. VRRS enables the collection of reliable balance data, such as swing area, swing speed, and number of oscillations, for assessing static balance and postural control in children with DCD (48, 49). Gait changes during walking are crucial in the assessment and early diagnosis of DCD. These changes are identified by analyzing the kinematics (movement analysis of body parts), kinetics (changes in the center of gravity, muscle dynamics, and joint dynamics), and plantar dynamics (changes in foot pressure) during walking in children. Tang (50) developed a framework using coronal 2D video recordings and posture estimation that allows distance-based features derived from conventional video recordings (e.g., mean distance from the left shoulder to the right hip or from the right shoulder to the left hip or 2D distance) to be utilized for the quantitative assessment of gait in children with DCD. Additionally, motion capture technology can measure, track, and record the movement patterns of objects in three-dimensional space, making it suitable for gait assessment in children with DCD (51). By attaching reflective markers to the major joints of the child, the 3D motion capture system can accurately evaluate gait data, including step length and gait speed, during walking. This provides an objective foundation for analyzing gait changes in children with DCD (52–54).

Standardized scales such as MABC-2, BOT-2, and PDMS-2 are commonly used for assessing fine hand activities, and they have demonstrated good reliability and validity. Additionally, precision instruments can provide quantitative assessment of upper limb and finger parameters during fine motor activities. Li proposed a fine motor assessment system that utilizes two cameras to record children’s performance in fine motor tasks. This system also incorporates automatic algorithms for task localization and single-task assessment, enabling automated scoring of fine motor skills and effective assessment for children with DCD (55). Three-dimensional motion capture can also be used to assess upper limb and finger movements during fine motor tasks in children with DCD. Using three-dimensional motion capture, Biancotto (56) discovered that children with DCD demonstrated a greater angle of spread between the thumb and forefinger during reaching and grasping tasks, compared to typically developing children. They also exhibited slower, more visually dependent, and more variable movements. Writing is a crucial everyday activity for children, and there are notable differences in movement trajectory and font size between children with DCD and typically developing children (4). A study showed that writing skills can accurately determine DCD in 94.9% of cases (57), Standardized scales such as the Handwriting Proficiency Screening Questionnaire and the Detailed Assessment of Speed of Handwriting Scale can be used to assess children’s writing skills (4). There are several tests available for assessing writing skills, which typically evaluate the efficiency of the handwriting process by counting the number of letters written within a designated time frame, and assess the quality of writing by evaluating handwriting legibility (58). Examination of children’s handwriting, including handwriting legibility and handwriting-generated movement. The legibility of handwriting can be assessed by means of the Handwriting Legibility Scale (59), and the handwriting-generated movement can be assessed by means of the Digital Writing Tablet and 3D Motion Technology (60, 61). Furthermore, digital writing tablets combined with 3D motion technology can analyze spatial and temporal data to evaluate handwriting accuracy and production movements (60), Additionally, 3D motion technology can monitor upper limb movement patterns during tasks such as copying and dictation, providing analysis on handwriting legibility, writing efficiency, and movement parameters related to writing performance (61). 3D Motion Capture holds extensive and versatile applications in the functional assessment of children with DCD. It enables precise measurement of perceptual sensations, dynamic capture of abnormal movement patterns during children’s walking for accurate assessment of gait parameters, and analysis and quantification of fine motor movements in hand grasping. This method offers a notably more precise and quantitative assessment compared to other tools.

Performance and engagement in activities of daily living (ADLs) are evaluated using the DCD Daily Scale, which encompasses motor performance (the degree to which the child completes the activity), daily participation (the extent to which the child engages in the activity), and ADL learning (the time taken by the child to learn the activity compared to their peers). This assessment provides insight into the children’s challenges in performing ADLs, learning, and participation, as well as the interplay between these areas (62, 63). Social skills are assessed using the Social Skills Improvement System Questionnaire (64), while psychosocial skills are evaluated through the Strengths and Difficulties Questionnaire (65, 66). The SDQ is a psychosocial assessment that measures the strengths and weaknesses of children and adolescents aged 4–16 years in domains such as emotional symptoms, behavioral problems, hyperactivity/inattention, peer relationship problems, and pro-social behavior (65). The Child Participation Scale (67) and the Canadian Occupational Performance Measure (68) are employed to assess children’s participation skills.

#### 2.2.3 Assessment of environmental factors

Environmental factors play a significant role in influencing the participation of children with DCD in activities, and parents often report facing more barriers and less support within their environment (4, 69–71). Assessing these factors involves evaluating the family, school, and community settings (see Table 2).

In the family environment, it is important to assess the personality traits of family members, living conditions, lifestyle, exercise spaces, and parental attitudes and behaviors. Understanding these factors provides insights into how the family environment may influence the child’s participation. In the school environment, the assessment should consider the availability of space and facilities, classroom activities, and interactions. Some indicators of the school’s psychological environment also warrant consideration, including measures of school adjustment, peer relationships, and teacher-student relationships. Evaluating these elements helps determine the level of support provided by the school and identifies potential barriers to participation. In the community environment, it is essential to assess outdoor sports spaces, the availability of playmates, and other factors that contribute to the child’s participation in community activities. Understanding the community environment provides insights into the opportunities available for the child to engage in various activities. Environmental factors can also be assessed using tools such as the Participation and Environment Measure-Children and Youth. This scale collects data on participation levels and the extent to which environmental factors either support or challenge children’s participation. It provides a comprehensive assessment of the home-school-community environments and their impact on children’s participation in activities. By evaluating environmental factors, professionals can gain a better understanding of the support and barriers that children with DCD may encounter in their daily lives. This information can guide interventions and strategies to enhance their participation in various activities (69).

By conducting a thorough assessment of the children’s body functions and structures, activities and participation, and environmental factors, a comprehensive understanding of their functional abilities can be obtained. This assessment provides a clear and detailed picture of the children’s overall functional status, enabling the development of personalized rehabilitation goals and targeted interventions.

## 3 Rehabilitation for children with DCD

### 3.1 Physical therapy

#### 3.1.1 Postural control and balance training

Postural control plays a crucial role in the performance and development of gross motor skills (72). Postural control training for children with DCD includes many aspects of balance program training, core stability training, muscle strength training, and lower limb proprioceptive training. A study conducted by Zolghadr (73) implemented balance corrective exercise training for children with DCD. This training included tasks like side-to-side walking, reverse walking, standing with feet together, standing on one foot, postural correction, and planking. After 8 weeks of training, there was a significant improvement in the static and dynamic balance of the children with DCD.

Core stability training is particularly important as it helps prevent improper movement patterns by maintaining proper balance and posture during functional activities (74). Another study by Shahrbanian (75) focused on core stability training in children with DCD, which resulted in noticeable improvement in their reaction time, dynamic balance, and static balance. Balayi (74) conducted a combined core stability-hemisball training program to improve bilateral balance and coordination in children with DCD. The results showed that 8 weeks of this training significantly enhanced postural control, dynamic balance, and static balance.

In addition, muscle strength training is crucial in improving balance for children with DCD. Insufficient leg muscle strength and slow muscle force generation can limit their balance function, making it difficult for them to control their pelvis or execute other body movements in a timely manner. Therefore, muscle strength training is an essential component for improving balance (72). Fong (76) conducted resistance training on children with DCD, leading to improvements in muscle force and time to peak muscle power. This training effectively enhanced neuromuscular function, balance, and balance strategies in children with DCD.

Research suggests that balance dysfunction in children with DCD is related to impaired proprioception in the lower limbs (77, 78). Therefore, proprioceptive training can be utilized to improve balance and adjust postural control (79). Proprioceptive training includes repetitive somatosensory stimulation, somatosensory discrimination exercises, passive motor training, and active sensory-motor training with enhanced somatosensory feedback (80, 81), and individualized activities can be designed to target proprioception improvement based on each child’s skill level.

Furthermore, it has been proposed that light touch (LT) feedback provides additional information to the central nervous system about body sway and spatial orientation. This feedback triggers more stable postural control mechanisms to maintain body balance (82), Therapists can incorporate LT to promote immediate postural stabilization during balance training. Additionally, Chen (83) discovered that finger dipping can serve as an effective rehabilitation strategy to enhance sensitivity to light touch in children with DCD. This enhances the impact of LT on improving body sway in children with DCD.

#### 3.1.2 Aquatic therapy

The aquatic environment possesses unique properties such as buoyancy, turbulence, hydrostatic pressure, and resistance, which can effectively improve gross motor skills in children with DCD (84). The buoyancy of water reduces joint loading and enables anti-gravity movements, thereby enhancing trunk stability. The viscosity and resistance properties of water provide additional resistance, helping to build muscle strength (85). Additionally, aquatic therapy (AT) enhances tolerance to multi-sensory stimuli, promotes blood circulation through hydrostatic pressure and temperature stimulation, increases joint range of motion, and reduces muscle spasms (86). Furthermore, the water environment offers a safe setting for children to practice motor strategies and specific tasks. In water, children are more willing to attempt tasks and skills that they may avoid on land (87, 88). The Halliwick Method is a specific AT intervention designed for individuals with motor dysfunction. It is based on principles of hydrostatics, hydrodynamics, and body mechanics (89).The method consists of four phases: adaptation to the water, rotation, control of movement in the water and aquatic locomotion. It incorporates exercises focusing on breath control, floating unaided, balance, lateral, sagittal, and longitudinal control, rotational control, upward thrusting skills, and swimming strokes (86). During the learning process, children engage with each other, promoting social interaction and respiratory control. This interaction positively impacts joint mobility, stability, muscular strength and endurance, muscle tone, normalization of involuntary motor responses, control, gait, and psychological adaptation to water (86). In a study by Hillier (87), AT implemented for children with DCD effectively improved their gross motor skills. In clinical practice, training programs can be individualized to cater to the unique needs of each child, taking into account their learning styles, physical activity, skill levels, and psychological and emotional characteristics.

#### 3.1.3 Kinesio tape

Kinesio tape (KT) is used to restore proper muscle function by stimulating the skin with the tape. When used as an aid during training, KT enhances muscle activation, postural control, and functional activity (90). In a study by Yam (90), KT was applied to the bilateral rectus femoris and gastrocnemius muscles in children with DCD. This resulted in improved dynamic balance performance, increased peak activation of the rectus femoris muscle, and prolonged time to peak muscle activation in specific extension directions. Another study by Li (91) investigated the effects of different KT patching methods in children with DCD, including no patching, gastrocnemius patching, tibialis anterior patching, and peroneus longus patching. The study found that muscle activity was significantly enhanced with the use of KT. Gastrocnemius patching improved balance during anterior–posterior swing, while tibialis anterior and gastrocnemius patching improved balance during medial-lateral swing. Additionally, KT facilitates improvement in proprioception. It provides mechanical pressure and stretch to the skin, stimulating skin mechanoreceptors and providing information about joint position and movement (91, 92).The repetitive stimulation enhances proprioceptive function in the body.

### 3.2 Occupational therapy

In occupational therapy (OT), the therapist assesses the child’s performance in activities of daily living, analyzes how the illness impacts occupational performance, and evaluates how the child’s environment influences their abilities (41). Based on this assessment, the therapist then selects suitable interventions for the child. In the case of children with DCD, OT primarily aims to improve fine motor skills, perceptual abilities, and executive functions.

#### 3.2.1 Fine motor training

Fine motor skills refer to the coordinated movements of the hand and the small muscle groups in the fingers and other parts of the hand (93). These skills involve various abilities, such as visual cues, tactile perception, and internal representation, and are crucial for children’s daily activities like picking up objects, grasping, manipulating, and tying shoelaces. The coordination of the small muscle groups in the hand serves as the foundation for precise movements. Improving hand strength and coordination can be achieved through exercises using grip strength devices, finger separation training, and two-hand coordination training. It is also important to pay attention to strengthening the proximal muscles, such as the shoulders and elbows, as they provide a stable base of support for distal control of the wrists, thumbs, and fingers, allowing the hand to be properly oriented in space and providing support and control of its movements (94).

Studies have shown that children with DCD often experience proprioceptive dysfunction in the shoulder, elbow, wrist, and hand joints, which contributes to their fine motor difficulties (95–97). Therefore, it is crucial to incorporate proprioceptive training for various upper limb joints. This can be achieved through stretching and weight-bearing exercises, such as static weight-bearing, upper limb balance exercises, dynamic stabilization exercises using a ball scapula, pinching activities, and active finger pinching tasks with or without elastic resistance (98–100). Additionally, elastic KT can be used to stimulate the local skin mechanoreceptors and proprioceptors in the surrounding tissues, thereby improving proprioception (92).

#### 3.2.2 Perceptual training

Visual and tactile perceptual skills training are crucial components of perceptual training for children with DCD. Visual feedback plays a significant role in the development of motor skills in children (101). The integration of visually perceived information into motor commands is essential for making adjustments to force output and correcting errors (102). Additionally, having good oculomotor control is important for accurately perceiving visual information (103). Eye movement control training incorporates various methods, such as visual training and quiet eye training (QET). Coetzee (104) implemented visual training in 32 children with DCD, combining perceptual and motor activities (balance, hand-eye coordination, bilateral integration, and vestibular integration) with visual exercises to enhance oculomotor control. After 18 weeks of treatment, significant improvements in oculomotor control skills, including visual tracking, fixation, alignment, and vergence, were observed in the children. QET involves maintaining fixation or tracking gaze on an object before initiating a critical action. It has been shown to effectively improve visuomotor control and attentional control (105). Norouzi (106) used QET with children with DCD to help them maintain visual attention and visuomotor coordination during coordination tasks, leading to enhanced bilateral arm coordination. Similarly, Rafique (103) implemented QET during a throwing and catching activity in children with DCD. They were instructed to fixate on the target position on the wall before throwing and then track the ball before catching it. This intervention effectively improved the visuomotor skills of the children, increased their concentration prior to throwing, and enhanced their anticipation and tracking ability. In addition to eye movement control training, engaging in sporting activities such as bowling, golf, cycling, football, skateboarding, and skiing have been found to improve physiological arousal, motor control skills, and expand visual attention in children with DCD (107).

Tactile perception also plays a crucial role in the daily activities of children with DCD, and improving tactile perception can enhance overall somatosensory functioning and fine motor control (108). Wang (38) implemented a tactile perception training program for children with DCD, where they matched objects based on six perceptual dimensions: texture, shape, hardness, size, weight, and contour. Following the training, significant improvements were observed in fine motor control tasks, fine motor integration, accuracy, manual dexterity, as well as visual perceptual functions such as visuospatial relations, visual memory, and visual sequential memory.

#### 3.2.3 Executive function training

Children with DCD often experience deficits in three key executive functions: inhibitory control, working memory, and cognitive flexibility (86).Engaging in different ball sports and judo training can be beneficial for training these executive functions in children with DCD. In ball games, such as returning a randomly thrown ball from an opponent, individuals need to pay close attention to the situation, encode visual cues, and inhibit inappropriate actions or responses. This type of training can be effective in improving children’s attentional, reactive, and inhibitory controls (109). Tsai (109) conducted a study where children with DCD were trained in table tennis, the game requires quick planning based on actual visual information and executing appropriate actions or inhibitory responses. The findings revealed that 10 weeks of table tennis training led to significant improvements in inhibitory control, cognition, and motor skills in children with DCD. On the other hand, judo training involves practicing various falling, throwing, and grappling techniques in unpredictable environments, which demand timely visuospatial cues (such as joint positions and movement patterns) to anticipate and react to an opponent’s actions. This type of training enhances working memory capacity, particularly in visuospatial working memory (110). Additionally, judo training involves interference or response inhibition, as participants need to exercise self-control when facing an opponent during judo matches. Therefore, judo training can effectively improve children’s inhibitory control skills (111).

### 3.3 Cognitive orientation training

Numerous studies have highlighted DCD as a motor cognitive disorder (112, 113). It is well-established that children with DCD exhibit deficits in various aspects of movement anticipation, basic motor learning processes, and cognitive control, all of which significantly contribute to their difficulties in performing daily activities (3). Consequently, implementing cognitive-based strategies such as cognitive orientation to daily occupational performance (CO-OP), motor imagery and action observation (MI + AO) therapy can effectively enhance children’s memory, attention, and mental planning skills, thereby improving their overall occupational performance.

#### 3.3.1 Cognitive orientation to daily occupational performance

Cognitive orientation to daily occupational performance (CO-OP), as an approach, aims to enhance task-specific performance through the use of cognitively based strategies. This approach emphasizes gaining a comprehensive understanding of task requirements, acquiring problem-solving strategies, and identifying crucial aspects of performance. By focusing on cognitive aspects rather than direct barriers that restrict task performance, CO-OP facilitates the development of transferable strategies that can be applied to various tasks (114). CO-OP interventions offer children the opportunity to choose their objectives, which helps maintain their motivation and engagement throughout the therapy process. Additionally, conducting CO-OP sessions in small groups fosters a cooperative environment where children can act as both learners and mentors. During therapy, the therapist initially teaches the child a comprehensive cognitive strategy known as Goal-Plan-Do-Check. This strategy involves setting action goals, developing action plans to achieve those goals, executing the plans to complete the goals, and evaluating the results. Following this, the therapist guides the child through holistic strategies to identify specific strategies for solving the task at hand. These guiding techniques may include reinforcement, providing stimuli to provide feedback to the child, modeling or demonstrating the skill, and offering prompts to support the child’s discovery of successful strategies. Gradually, the prompts are reduced once the child has successfully completed the task with an improved plan (115, 116). In Thornton’s study (68), a group of children with DCD participated in a 10-week CO-OP intervention. The results showed that children in the CO-OP group demonstrated improvements in impairment, participation, and activity levels.

Similarly, Araújo’s study (116) investigated the effects of a 6-week CO-OP program involving both children with DCD and their parents. The study found that all the children showed improvements in their occupational performance on the goals they selected after receiving the CO-OP treatment. Furthermore, five of the children were able to apply the cognitive strategies they learned to tasks that were not specifically addressed in the treatment. CO-OP interventions have been proven effective in enhancing children’s task-specific performance by targeting cognitive strategies related to their chosen goals. Consequently, this approach leads to improvements in their overall activities and participation levels. Additionally, CO-OP facilitates the application of holistic or domain-specific strategies to other aspects of daily life as children’s cognitive strategies are enhanced.

#### 3.3.2 Motor imagery and action observation (MI + AO)

MI therapy, which is a cognitive-based intervention, has also been shown to effectively improve motor planning in children with DCD (117, 118), MI involves mentally rehearsing or simulating movements from a first-person perspective, without physically executing the movements. Through MI, children can develop accurate internal models that aid in motor planning, activating the brain regions responsible for planning and executing motor skills (36, 117, 119, 120). Hoyroo (36) conducted a study to investigate the impact of MI on grip choice strategies in children with DCD. The participants, including both children with DCD and typically developing children, were engaged in a grip choice task with various color sequences. The MI group was instructed to mentally visualize the instructions for completing the movements before executing the task. The results indicated that children with DCD in the MI group demonstrated significantly improved motor planning abilities across all color sequences.

MI is frequently combined with AO in the treatment of motor planning difficulties (121). AO involves observing another individual performing a specific action to obtain precise visual and temporal motion cues. This visual information is then mapped onto a motion circuit and utilized to generate motion imagery that is closely related to the observed action (122). Both MI and AO activate brain regions that are involved in motor planning and execution, as they share neural networks associated with internal modeling processes (3, 121). Numerous studies have demonstrated that combining MI and AO can lead to improvements in motor skills and activities of daily living in children with DCD (123). In the Marshall study (124), children with DCD were divided into two groups: the MI + AO group (watching a video of the task while mentally imagining the sensations associated with performing the action) and the control group (watching a video unrelated to movement). During a visuomotor rotation task, the children in the MI + AO group demonstrated faster completion times, more goal-oriented eye movement patterns, and smoother movements. This study highlights the potential of combining MI and AO techniques in training for motion planning to enhance the development of internal models.

### 3.4 Virtual reality

In recent years, the use of virtual reality (VR) has become increasingly prevalent in the rehabilitation of children with DCD, thanks to advancements in technology. VR offers a simulated experience of real-life environments, providing an enjoyable and accessible platform for children to enhance their confidence in their own abilities. This positive cycle of reinforcement helps to motivate children in overcoming challenges they face in their daily lives. Engel-Yeger’s study (125) illustrates how VR-based exercises and virtual games are used to provide enjoyable and successful experiences for children with DCD. These activities allow children to simulate the actions of their typically developing peers, ultimately leading to improvements in real-world activities and social participation skills.

Compared to traditional sedentary video games, virtual games are considered active video games that involve larger scale body movements in an interactive environment. An example of an active video game for VR systems is Wii Fit training. This game utilizes handheld controllers or balance board devices to track limb movements and translates this information into controlling avatars within a virtual gaming environment (107). In the Hashemi study (107), it was found that children with DCD who participated in 8 weeks of Wii Fit training experienced noteworthy enhancements in various aspects of visual perception, such as visual discrimination, visual memory, visual perception, visuospatial relations, visual form constancy, visual sequence memory, visual graphic ground, and visual closure. Improvements were also observed in executive function tasks, including planning, attention, simultaneous processing, and sequential processing. Furthermore, VR technology can be effectively utilized in AO and MI training. Through the use of VR, participants are able to perceive the virtual environment as if it were real and engage in interactive body movements to participate in the game or training session (126). VR offers a multitude of sensory feedback options, guiding the child’s attention towards external effects and motor outcomes. This in turn facilitates improved predictive motor control for children with DCD and enhances their ability to visualize and execute motor tasks (127).

### 3.5 Intervention of environmental factors

Environmental factors are of utmost importance in the motor development of children with DCD. Creating an environment that is conducive to motor development allows children to consistently practice and enhance their motor skills. Therefore, interventions should not only focus on individual therapy but also include modifications to the child’s home, school, and community environments. These modifications aim to support the acquisition of motor skills and promote social participation among children with DCD. This entails providing suitable and sufficient space within the home, school, and community environments to accommodate various physical, cultural, and recreational activities. It is vital to encourage the active involvement of parents and peers in a wide range of activities that foster the motor development of children. Furthermore, the influence of electronic screens on children’s motor abilities cannot be disregarded as it becomes more prevalent. Research has shown that early exposure to electronic screens has a detrimental effect on children’s exercise duration, neuromusculoskeletal development, and motor skills (128–130). As a result, strict limitations should be implemented, ensuring that children’s exposure to electronic screens does not exceed 2 h per day.

Ecological intervention is a highly effective treatment for children with DCD. This approach not only focuses on restoring the individual’s motor function but also addresses environmental factors that contribute to DCD. These factors include the family, school, and community, all of which are considered essential components of the intervention (69). For example, home-based interventions during weekends can be implemented, involving parents actively in the treatment process. This can include improving parenting styles, providing encouragement and support to their children. In addition, peer-mediated physical activities can be introduced in school and community settings. These activities aim to promote the practice and acquisition of new motor skills in various activities, as well as enhance children’s social participation and ability to perform daily tasks. Ferguson conducted a nine-week health promotion program for children with and without DCD in a school setting, working to create child-friendly health conditions in the school and to influence the health-related knowledge and behaviors of children, educators, and parents, which resulted in improvements in the athletic performance of children with DCD as well as in their level of physical fitness (131).

## 4 Conclusion

Given the diverse impairments and limitations experienced by children with DCD, it is crucial to conduct a thorough assessment of their body functions and structures, activities and participation, and environmental factors. These assessments help to determine the extent and severity of the impairment of children’s body functions and structures, to identify the extent to which their activities and participation are limited, and to understand the environmental factors that hinder or promote them. Based on the assessment results, individualized rehabilitation goals can be established, and specific interventions can be formulated to address the unique needs of each child. Rehabilitation for children with DCD encompasses various interventions (Figure 3), such as physical therapy (PT), OT, cognitive orientation training, the use of emerging technologies like VR and the environmental factors. PT aims to reduce impairments, while OT focuses on enhancing limited activities and participation in daily life. However, it is important to note that improvements in impairments do not always lead to improvements in daily activities and participation (4, 132). Therefore, a multidisciplinary approach is often employed to address both impaired functioning and limited activities and participation in children with DCD.

In addition to other aspects, OT emphasizes family-centered therapy as a principle. It emphasizes the involvement of parents in their children’s treatment (132). Collaboratively, therapists and parents establish therapy goals, which can be related to education, self-care activities, or social interaction. Interventions are tailored to the specific abilities and needs of the child (41).Cognitive orientation training holds significant importance in the rehabilitation of children with DCD, as it is recognized as a motor cognitive disorder in numerous studies (112, 113). By acquiring problem-solving strategies at a cognitive level, children can enhance task-specific performance and improve their activities and participation. Moreover, the ability to generalize these strategies to other tasks is crucial (116). In this context, the use of emerging VR technologies offers enhanced and practical movement environments for the treatment of children with DCD, presenting a promising therapeutic approach. The treatment should also address the impact of environmental factors on the child’s functioning in society, their ability to perform actions or tasks, and their overall physical and mental well-being. The intervention of environmental factors encompasses all aspects of the family, school, and community environments. Throughout the treatment process, it is important for parents, teachers, and other individuals in the child’s surroundings to provide the necessary support and create a favorable external environment. By working together, a conducive environment can be established that facilitates the children’s acquisition of motor skills and enhances their social participation.

Regardless of the specific approach, intervention strategies for DCD can generally be classified into two main categories (133).The first category involves the application of activities aimed at addressing the underlying behavioral issues. This approach, known as the bottom–up process-oriented approach, focuses on reducing impairments and improving the child’s body functions and structures. The second category focuses on directly addressing the behavioral issues themselves. This approach, referred to as the top–down task-oriented approach, emphasizes changes at the level of activities and participation (For a categorization of the intervention strategies mentioned in this paper, see Table 3). In clinical practice, it is crucial to consider the generalizability of intervention effects to the different environments in which a child engages in daily activities and participates. Therefore, integrated interventions that focus on tasks are highly recommended for DCD. These interventions enable children to acquire problem-solving skills, enhance their motor planning and execution abilities, and apply the intervention’s effects to more complex everyday activities.

**Table 3:** Clinical rehabilitation interventions.

	Rehabilitation interventions	
	Process-oriented approach	Task-oriented approach
Physical therapy		
Postural control and balance training		
Balance program training	Balance corrective exercise training	
Core stability training		Core stability-hemisball training
Muscle strength training	Resistance training	
Limb proprioceptive training		Personalized proprioceptive stimulation activities
Aquatic therapy		aquatic training activities
Kinesio tape		
Occupational therapy		
Fine motor training		Coordinated activity training of the proximal and distal muscles of the upper limb; Upper limb joint proprioceptive training activities
Perceptual training		
Visual perceptual		Visual training Quiet eye training Sporting activities (bowling, golf, cycling)
Tactile perceptual	Tactile perception training program	
Executive function training (Inhibitory control; Working memory; Cognitive flexibility)		Ball sports; judo training
Cognitive orientation training		Cognitive orientation to daily occupational performance; motor imagery and action observation
Virtual reality		Active video games
Environmental factor interventions		

## Author contributions

JG: Writing -original draft, Writing -review & editing. WS: Writing -original draft, Writing - review & editing. YZ: Writing -original draft, Writing -review & editing. DH: Writing -review & editing. JW: Writing -review & editing. AZ: Writing -review & editing. XK: Writing -review & editing.
